# Biopolymer Composites: Synthesis, Properties, and Applications

**DOI:** 10.3390/ijms23042257

**Published:** 2022-02-18

**Authors:** Ana M. Díez-Pascual

**Affiliations:** Universidad de Alcalá, Facultad de Ciencias, Departamento de Química Analítica, Química Física e Ingeniería Química, Ctra. Madrid-Barcelona, Km. 33.6, 28805 Alcalá de Henares, Madrid, Spain; am.diez@uah.es

Petroleum-based plastics can be found everywhere in our habitual life in diverse applications such as automobiles, aerospace, and medical science. However, apart from the economic field, most petroleum-driven materials induce an unsustainable environment. In this regard, environmentally friendly, biodegradable, and nontoxic materials, especially from renewable resources, have attained great levels of attention, and a strong effort has been focused on research on biodegradable and biocompatible polymers to replace petroleum-based commodity plastics [1,2]. Biopolymers are polymers synthesized from natural sources: either chemically synthesized from a biological material or completely biosynthesized by living organisms. However, biopolymers frequently display poor mechanical properties and restricted processing capability and end-use application. In order to overcome these drawbacks and develop advanced materials for a broad range of applications, biopolymers can be reinforced with fillers or nanofillers [3]. Composites based on biopolymers are named as “green composites”. They can be degraded by the act of environmental factors such as air, light, heat, or microorganisms. In a biopolymer composite, the biopolymer matrix mainly rules structure, environmental tolerance, and durability, while reinforcement conditions the stiffness and strength of the composite [4,5]. Value-added new applications of biopolymer composites guarantee possible developments in international marketplaces. The efforts to develop environmentally friendly composite products with better performance have attained some foremost worldwide applications and are still ongoing. This Special Issue, with a collection of eight research articles, offers representative examples of the most recent advances in the synthesis, properties and applications of biodegradable biopolymer composites. Attention has been focused on production, processing, and application of biodegradable composites prepared from polymers such as polylactic acid (PLA) [6], chitosan [7], alginate [8], natural rubber [9], and so forth, which are amongst the most promising matrices for green composites in the future.

One of the most widely used biopolymers is PLA, a fully biodegradable and biocompatible polymer widely used in packaging, agricultural, personal care, cosmetic, biomedical, and tissue engineering sectors [10]. Many researchers have explored bactericidal films based on PLA with the addition of different reinforcements such as plant tree oils [11,12]. An innovative reinforcement that can impart antibacterial properties is birch tar (BT), a typical product of the distillation of wood and birch bark. The chemical components of BT are mostly phenol derivatives (guaiacol, creosote, and pyrocatechin), organic acids, and resin substances. Thus, biopolymer composites including PLA, poly(ethylene glycol) (PEG) as plastizer (5 wt%), and BT (1, 5 and 10 wt%) have been developed and characterized [6]. PLA film with 10 wt% BT displayed the best antibacterial effect against plant pathogens, i.e., *X. campestris*, *A. tumefaciens*, *P. corrugata*, and *P. syringae*. The addition of BT results in a material with biocidal effects and advantageous physicochemical and structural properties for agricultural and horticultural applications.

Another alternative to develop antimicrobial nanomaterials for agricultural applications is based on natural polysaccharides such as arabinogalactan (AG), arabinogalactan sulfate (AGS), and κ-carrageenan (κ-CG) [13]. In this regard, the effect of a trace element, Mn, on the physicochemical properties of the composites was evaluated. The results obtained demonstrate that these nanocomposites can be used as safe and biodegradable carriers of mineral trace elements possessing biologically active properties for plant protection, for instance, against Gram-positive bacterium such as *R. erythropolis*.

Other important polysaccharide-based matrices include alginate, which is widely used to developed hydrogels used for cellular applications and drugs. Cross-linked alginate hydrogels are used in many fields, including waste removal agents, drug carriers, wound dressing materials, food products, and tissue engineering and so forth. In addition, alginate is one of the biofilm substances produced by bacteria, and alginate hydrogels are used as a model biofilm in laboratory research [14]. In this regard, alginate hydrogels containing rhamnolipids, a group of biosurfactants produced by many microorganisms such as pathogen Pseudomonas aeruginosa, have been used to investigate the existence of a relationship between rhamnolipids and bacterial biofilm [8]. The presence of rhamnolipids changes the mechanical properties of the alginate: at concentrations below the CMC, the addition of this biosurfactant decreases compression loads, while it increases at concentrations above CMC.

Another polysaccharide widely used in biomaterials as it may be isolated from food industry byproducts is chitosan [15]. It is safe and nontoxic; thereby, it may have contact with human tissues [16]. The foremost drawback of chitosan is its low stability; hence, cross-linkers are required to improve its properties [4]. Polyphenols such as tannic acid, gallic acid and ferulic acid are able to form strong hydrogen bonds with polymers; thereby, they are considered as effective cross-linkers for polysaccharides [7]. Polyphenols provided effective antimicrobial activity; hence, chitosan/phenolic acid-based materials have potential applications in food technology, as encapsulating agents, biomaterials, bioadsorbents, or coatings.

Packaging materials must meet requirements, such as non-toxicity, water vapor and oxygen impermeability, transparency, and good mechanical properties. Previously, synthetic antioxidants, such as butylated hydroxyanisole (BHA) or butylated hydroxytoluene (BHT), were widely applied in food packaging materials to avoid lipid oxidation. Nonetheless, currently, they are substituted by compounds such as polyphenols, plant extracts, or essential oils [17,18]. A recent study has assessed the antioxidant capacity and stabilization efficiency of cannabidiol (CBD) extracts from cannabis plants in two biopolymers, PLA and ethylene–norbornene copolymer (Topas), that are widely used in packaging materials [19]. The addition of CBD as a natural additive results in an increase in oxidative-induction time/temperature. In addition, this additive can also be considered as an aging color indicator, which allows controlling changes that occur during the lifetime of the polymeric products.

A group of polymers with very versatile properties is polyurethanes, which are obtained by the formation of urethane bonds due to the reaction of polyols and polyisocyanates [20]. The use of properly selected polyols and polyisocyanates allows obtaining polyurethane materials in various forms, including elastomers, coatings, adhesives, rigid and flexible foams, films, and fibers. As a result, polyurethanes have been widely used in many fields, such as thermal and electrical insulation and coatings, biomedical applications, construction, high-performance adhesives, the footwear market, and packaging or furniture [21]. In a novel study, ground plum stones and salinized ground plum stones were used as natural fillers for polyurethane composite foams. The influences of 1, 2, and 5 wt% of fillers on cellular structure; foaming parameters; and mechanical, thermomechanical, and thermal properties of manufactured foams were evaluated [22]. The results showed that the morphology of the biocomposites is affected by the type and content of filler. In addition, the modified polyurethane foams showed enhanced mechanical properties, such as higher compressive and flexural strength, improved thermal stability, and hydrophobic character.

Cellulose nanofibers (CNF) isolated from plant biomass have also attracted considerable benefits in polymer engineering. The restrictions associated with CNF-based nanocomposites are related to time-consuming preparation methods and the absence of desired surface functionalities [23]. On the other hand, ZnO nanoparticles display outstanding properties including good antibacterial properties, mechanical strength, low coefficient of thermal expansion, and high thermal conductivity and are great potential candidates for biopolymers for applications in medicine and food industries [24,25,26]. In a novel investigation, the feasibility of preparing multifunctional CNF-ZnO nanocomposites with dual antibacterial and reinforcing properties via a facile and efficient ultrasound route has been reported [27]. The nanocomposites showed improved thermal stability compared to raw CNF and inhibitory activities against Gram-positive *S. aureus* and Gram-negative *S. typhi* bacteria. A CNF-ZnO-reinforced natural rubber nanocomposite film, manufactured through latex mixing and casting methods, showed 42% improvement in tensile strength compared with the neat rubber. These novel nanocomposites are perfect candidates for use in the biomedical field and in the development of high strength rubber composites.

On the other hand, different herbs such as peppermint, German chamomile, and yarrow have been used as natural fibers to create natural rubber-based biocomposites [9]. Rubber is one of the most important elastomers in terms of versatility and application level due to its superior elasticity, resilience, and abrasion resistance. Compared to manmade fibers such as glass or carbon, natural fibers have so many advantages such as natural abundance, low cost, low density, competitive specific mechanical properties, reduced energy consumption, carbon dioxide (CO_2_) sequestration, biodegradability, etc. The developed rubber/fiber composites showed improved barrier and mechanical properties. Moreover, an increase in the cross-linking density of the materials before and after the simulated aging processes, compared to the reference sample, was observed. Overall, these composites are expected to play very important roles in our industries and in our day-to-day life requirements.
